# How specific are affective and attachment instability to adolescents with non-suicidal self-injury? A comparison with adolescents with major depression

**DOI:** 10.1186/s40479-026-00336-4

**Published:** 2026-02-12

**Authors:** Annekatrin Steinhoff, Julian Koenig, Julia Blanke, Philip Santangelo, Ulrich Ebner-Priemer, Michael Kaess

**Affiliations:** 1 University Hospital of Child and Adolescent Psychiatry and Psychotherapy, University of Bern, Bolligenstrasse 111, 3000, Bern, 60 Switzerland; 2 Department of Child and Adolescent Psychiatry, Psychosomatics and Psychotherapy, University Hospital Cologne, University of Cologne, Cologne, Germany; 3 Kinder- und Jugendpsychiatrische Institutsambulanz, Zentrum für Psychiatrie Calw, Klinikum Nordschwarzwald, Calw, Germany; 4 Department of Behavioural and Cognitive Sciences, University of Luxembourg, Esch-sur- Alzette, Luxembourg; 5 Institute of Sport and Sports Science, Karlsruhe Institute of Technology, Karlsruhe, Germany; 6 Department of Child and Adolescent Psychiatry, Center for Psychosocial Medicine, University Hospital Heidelberg, Heidelberg, Germany

**Keywords:** Affective instability, Non-suicidal self-injury, Adolescents, Ecological momentary assessment, Borderline personality disorder

## Abstract

**Background:**

Affective and interpersonal instability are suggested to underlie adolescent non-suicidal self-injury (NSSI). However, previous research has not directly compared adolescents with NSSI to those with psychiatric conditions that are often, but not always, correlated with NSSI. We explored the specificity of affective and interpersonal instability to adolescent NSSI compared to depression.

**Methods:**

Based on a clinical sample of 70 adolescents (56 female and 14 male), we compared a group with NSSI (*n* = 50) with a clinical comparison group diagnosed with major depression and no engagement in NSSI (*n* = 20). We used high-frequency ecological momentary assessments (EMA) of affect and interpersonal attachment over four days. Mean affect and mean attachment as well as the root mean squares of successive differences (RMSSD) indicating instability of affect and attachment were calculated. The groups were compared using regression modeling.

**Results:**

Adolescents with NSSI reported less positive affect and lower levels of attachment to their mothers and best friends than those with depression. Moreover, models adjusting for gender and mean affect and attachment, respectively, indicated that the NSSI group experienced more affective instability (*b* = 6.26, 95% CI = 0.79—11.73, *p* = 0.025). The two groups experienced similar average levels of attachment instability. Although there was no moderating effect of gender in the reported associations, the link between affective instability and NSSI was mainly driven by the male subsample, whereas low levels of attachment were associated with NSSI especially in females.

**Conclusions:**

Affective instability is especially high among adolescents with NSSI. When co-occurring with other disorders, NSSI may serve as an identifier of a patient’s particularly strong need for treatment aiming at affect stabilization.

**Supplementary Information:**

The online version contains supplementary material available at 10.1186/s40479-026-00336-4.

## Background

Non-suicidal self-injury (NSSI) refers to the deliberate infliction of harm upon one’s own body tissue without the intent to die and for purposes that are not socially or culturally sanctioned [1]. In clinical adolescent samples, the prevalence of NSSI typically ranges around 60% [2], with up to 78% of adolescents with NSSI fulfilling DSM-5 (section three) diagnostic criteria for the proposed NSSI disorder (NSSID) [3]. Common self-reported functions of NSSI include intrapersonal functions, especially the regulation of intense negative affect, and interpersonal functions, such as the expression of distress [4]. Evidence suggests that, although NSSI may be a stand-alone condition in a few cases, it is, overall, a transdiagnostic marker of emotional and interpersonal dysregulation [5–8].

Emotional and interpersonal dysregulation, including in the forms of severe mood impairment and affective and interpersonal instability—which are also core features of borderline personality disorder (BPD) [9–11] and the proposed dimensional borderline personality pathology [12]—may be ‘driving forces’ behind NSSI. Adolescents affected are likely to perceive their marked levels of negative affect and poor attachment as unbearable, their sudden and frequent changes of affect and attachment experiences as unpredictable, and NSSI as a powerful method to regulate themselves and regain a sense of control [13, 14]. However, since NSSI co-occurs with a variety of psychiatric disorders in the realm of personality pathology and mood disorders [8, 15, 16], it is unclear whether or to what extent NSSI may be a specific identifier of affective and attachment instability. Comparisons of emotional and interpersonal functioning related to NSSI versus other (frequently comorbid) clinical conditions could make important novel contributions to the development and differentiation of diagnostic criteria and help identify relevant treatment targets in individualized therapy. Yet the large overlap of NSSI and other conditions render comparisons between clinical adolescent samples with and without NSSI difficult to achieve, especially when sample sizes are small [17]. To provide new insights into the specificity of emotional and interpersonal dysregulation in NSSI, targeted recruitment of adolescent patients without NSSI is necessary.

To facilitate ecologically valid assessments of the links between NSSI and emotional and interpersonal dysfunction, researchers have increasingly implemented innovative methodologies involving real-time repeated data collection over the day, such as ecological momentary assessments (EMA). Data collection using EMA involves the administration of brief questionnaires several times per day, for example using a smartphone-based application, which enables participants to report on real-life experiences while being in their home environment [18, 19]. Studies using these methods revealed that negative and unstable (i.e., rapidly changing) affect and poor and unstable interpersonal attachment are linked with NSSI [17, 20–22]. Furthermore, evidence suggests that patients with BPD, which often—but not always—involves NSSI, have uniquely high levels of affective instability [10]; and among adolescents with NSSI, the number of BPD symptoms was associated with more instability in affect and attachment [17]. Although these findings could indicate that NSSI and other BPD symptoms are manifestations of the same underlying dimensional pathology [12], it is important to note that other research suggests that affective instability may be best understood as a transdiagnostic phenomenon [23, 24]. Indeed, it has been associated with a variety of psychiatric impairments, including mood disorders [25], which are, in turn, frequently associated with adolescent NSSI [5]. Patients with depression—and without NSSI—thus constitute a particularly important clinical comparison group for research in the specificity of affective instability to adolescent NSSI.

Notably, to our knowledge, no previous study has compared affective and attachment dynamics in NSSI versus specific psychiatric disorders in adolescence, when NSSI and borderline personality pathology typically emerge [26]. Indeed, severity of borderline pathology typically increases during adolescence, and a better understanding of its features during the adolescent period could help advance early identification of affected individuals, improve developmentally adapted interventions, and facilitate timely and effective prevention of the normative increase of the pathology [27]. Findings based on adult samples may not be generalizable to the adolescent population, since patterns of NSSI and BPD are partly age specific. For example, NSSI commonly declines from late adolescence until young adulthood [28, 29], whereas borderline pathology commonly increases until the early twenties and then decreases, associated with marked changes in its symptomatic presentations [30]. Relatedly, the specificity of NSSI as a symptom of BPD likely changes with increasing age. Although NSSI is a very common symptom of BPD across age groups [31], it can be considered a transdiagnostic marker of mental disorders, and even psychological strain without mental disorder, especially among adolescents [5]. Considering the differential role of NSSI in adolescent versus adult populations, a better understanding of age-specific underlying psychological traits is of high clinical relevance.

There is also a paucity of knowledge about gender differences in the links between NSSI and affective and attachment instability, and NSSI in general. Clinical research on NSSI has mostly been based on female samples, partly because the prevalence of NSSI is higher among female than male adolescents [3, 32]. However, the prevalence of NSSI is also considerable in male samples. For example, a meta-analysis of the epidemiology of NSSI across different age groups revealed a lifetime prevalence of 26% among males [33]. A recent community representative study showed that one in five male adolescents reported any NSSI between ages 13 and 20 years [29]. Thus, a better understanding of NSSI in gender-diverse samples and males is needed, especially because some evidence suggests that the functions, methods, and diagnostic correlates of NSSI may partly be gender-specific [32–35]. For example, the link between negative relationship experiences with parents and NSSI seems to be stronger in females than males [32].

### The present study

Our aim was to assess whether NSSI is uniquely associated with emotional and interpersonal dysfunction. To achieve this aim, we recruited and compared two groups of adolescents: one group including adolescents who had engaged in NSSI on five or more days during the previous year (i.e., NSSID criterion A) and the other group including adolescents who fulfilled DSM-5 criteria for major depression without engagement in NSSI. Given that affective and interpersonal dysregulation are conceived of as core features underlying NSSI, we first hypothesized that adolescents with NSSI report more negative affect and greater affective instability than depressed adolescents without NSSI. Second, we hypothesized that adolescents with NSSI show less interpersonal attachment and greater instability of interpersonal attachment than those without NSSI. Assuming that adolescents typically spend considerable time of their daily lives with their mothers (family context) or a best friend (peer context), we focused on attachment with these relationship partners as important representatives of the family and peer contexts. Finally, we explored whether differences between the NSSI and depression groups generalize across genders.

## Methods

### Participants

Participants were recruited from the Clinic for Child and Adolescent Psychiatry, Centre for Psychosocial Medicine, University of Heidelberg. Specifically, patients in the NSSI group were recruited from the specialized outpatient clinic for risk-taking and self-harming behaviors (Ambulanz für Risikoverhalten und Selbstschädigung: AtR!Sk). The main inclusion criterion for our study was NSSI incidents on a minimum of 5 days during the previous year (DSM-5 criterion A). In a previous study, a sub-sample of these patients has been included to compare affective and attachment instability in adolescent patients with NSSI to a *healthy* control group [17]. For the present investigation, we expanded the sample with NSSI and recruited an additional sample of patients with a clinical diagnosis of an affective disorder, a maximum of two BPD symptoms, and no indication of NSSID (i.e., a *clinical* comparison group). This group was recruited from the overall clinic (AtR!Sk and other outpatient centers and inpatient clinic).

### Procedures

Patients were invited to the clinic for a diagnostic interview, where they were also equipped with study smartphones and trained on using the EMA app (movisensXS from Movisens GmbH, Karlsruhe, Germany). The smartphones had to be returned after completion of the EMA, which was carried out over the following two weekends (i.e., four days). With this design, our study focuses on weekend-specific affective and attachment dynamics, precluding a perspective on school-related stressors. The participants were prompted 12 times a day starting at 10 a.m. and asked to fill out a set of questions. The prompting ended at 10 p.m. or, if the maximum number of 12 completed prompts had not been reached, at 12 a.m. at the latest. A random time-sampling schedule in 60 min intervals was used. Responses were recorded together with timestamps. More details on the procedure can be found elsewhere [17].

The study was carried out in accordance with the declaration of Helsinki (World Medical Association 2013). Ethics approval was obtained from the Ethical Committee of the Medical Faculty, Heidelberg University (ID S-448/2014). Patients and their legal caregivers provided written informed consent. Participants received a cash incentive (12 Euros for participating in a diagnostic interview, plus 1 Euro for each prompt that was answered during a subsequent EMA if at least half of the prompts were answered).

### Measures

#### Diagnostics at the lab visit

*NSSI* was assessed using the German version of the Self-Injurious Thoughts and Behaviors Interview (SITBI-G [36]. *BPD* was assessed using the German version of the Structured Clinical Interview for DSM-IV Axis II (SCID-II [37]. To assess *Axis I disorders*, the German version of the Mini-International Neuropsychiatric Interview for Children and Adolescents (MINI-KID 6.0 [38]) was used. In the clinical comparison group, the German version of the Child and Adolescent Depression Inventory was administered [39] using a questionnaire. It included 27 items (with codes 0 = absence of the symptom, 1 = medium severity, and 2 = high severity) and we created a sum score that represents the overall severity of depressive symptoms.

#### Affect and interpersonal attachment assessed with EMA

*Affect* was assessed using an adapted version of the Multidimensional Mood Questionnaire, which was designed specifically for the use in EMA and shows good psychometric properties [40]. Participants reported how they were currently feeling, using four bipolar items (unwell – well, agitated – calm, satisfied – dissatisfied, relaxed – tense). Answers were given on a visual analog scale from 0 to 100 and we reversed the coding of the two items assessing negative affect. Santangelo and colleagues [17] reported satisfactory reliability (*ω* = 0.84) of the four-item scale. We computed a mean score for each EMA prompt that was completed and then an overall mean score across prompts, which represents an individual’s average level of affect. Higher values indicate more positive affect.

*Interpersonal attachment* was assessed with four items addressing current attachment to, first, one’s mother and, second, one’s best friend [17]. The participants were asked (a) “how close do you feel to your mother/best friend right now,” (b) “how important is your mother/best friend to you right now,” (c) “what do you think, how close does your mother/best friend feel to you right now,” and (d) “what do you think, how important are you for your mother/best friend right now?” Answers were given on a visual analog scale from 0 to 100. Santangelo and colleagues [17] reported a reliability of *ω* = 0.76 for the four-item scale assessing attachment to the mother, and *ω* = 0.83 for attachment to the best friend. We combined the four items by computing mean scores for each prompt, and then computed the overall individual’s average of attachment to the mother and best friend, respectively. Higher values indicate stronger perceived attachment.

To assess *instability*, we used the root mean square of successive differences (RMSSD), which represents both the variability and temporal dependency of affect or attachment dynamics [41, 42]. To compute the scores, we, first, calculated squared successive differences (SSD) between adjacent assessments of each indicator of (a) affect, (b) attachment to mother, and (c) attachment to best friend, respectively. This was done for all adjacent responses with in-between intervals lasting 150 min or less [17]. Second, we square-rooted the SSDs and combined the four square-rooted SSDs per outcome to assess instability (i.e., RMSSD) of (a) affect, (b) attachment to the mother, and (c) attachment to the best friend. Higher values on the RMSSD variables indicate more instability in the specific domains.

### Statistical analyses

To test our hypotheses regarding group differences in affect and attachment, we specified linear regression models. Regarding the individuals’ mean levels of affect and attachment, we specified two models for each outcome. The first model included the dummy-coded group membership as the only predictor, and in the second model we added gender as a control variable. For the instability outcomes (i.e., RMSSD of affect and attachment), we also specified a third model, which adjusted for the individual mean level of affect or attachment, respectively. This was done considering that fluctuations of affect and attachment may vary as a function of respective base levels [43].

The number of individual observations may have an impact on the precision of the means and variances of our composite scores (e.g., the number of completed EMAs may impact the precision of an individual’s mean score of affect and RMSSD of affect). In our sample, the number of completed EMAs was lower in the NSSI group (*M* = 34.16, *SD* = 13.31) than in the depression group (*M* = 41.80, *SD* = 7.18; Cohen’s *d* for group difference = 0.64, *p* = 0.018). To correct for potential biases, we used analytic weights (i.e., the individual number of responses used in the mean score and RMSSD calculation), as done by Santangelo and colleagues [17]. In the main manuscript, we report the results from the analyses using weights; results from sensitivity analyses without using weights are provided in the supplementary material.

The final step was to explore gender differences in the associations between group membership and all outcomes. For formal hypothesis testing, we included interaction terms (gender*group) in the main models. In addition, we conducted the main models separately based on the male and female sub-samples. To determine significant associations, we considered 95% confidence intervals of unstandardized coefficients and two-tailed *p*-values, using an α-error probability of 5% as a reference. We carried out the analyses using Stata/SE 17.

## Results

Our sample included 70 adolescents. Of those, 56 were female and 14 were male. Table 1 provides an overview of sample characteristics by group. The NSSI group included *n* = 50 adolescents, and the comparison group included *n* = 20 adolescents with a mood disorder (mainly major depression). The NSSI and depression groups did not differ regarding the participants’ average age. The proportion of female participants was higher than that of male participants in both groups, especially in the NSSI group. Specifically, the NSSI group included 43 female and seven male adolescents, whereas the depression group included 13 female and seven male adolescents. Most participants were attending schools requiring medium or high achievement levels (Gymnasium and Realschule). For most participants in the depression group, the research team was able to confirm current affective disorders. The groups did not differ regarding the prevalence of most other diagnoses, except for substance use disorders and disorders of adult personality and behavior, which were more prevalent in the NSSI group. Thus, both groups were heterogeneous in terms of comorbidities, as expected in clinical samples and especially those with NSSI, which has been shown to be a transdiagnostic marker of psychopathology [5]. By design of our study, BPD was exclusively diagnosed in the NSSI group.

**Table 1:** Group characteristics

	NSSI (*n* = 50)	Depression (*n* = 20)	*p* for group difference
Age (years): M (SD)	15.78 (1.17)	15.40 (1.27)	0.234^a^
Gender: female (vs. male): *n* (%)	43 (86%)	13 (65%)	**0.047** ^b^
Type of school: *n* (%)			
Hauptschule	4 (8%)	2 (10%)	0.787^b^
Realschule	17 (34%)	5 (25%)	0.464^b^
Gymnasium	20 (40%)	11 (55%)	0.254^b^
Other school	5 (10%)	1 (5%)	0.500^b^
Not attending school	4 (8%)	1 (5%)	0.660^b^
Mental Disorders (*n*,%) and symptoms (M, SD)			
Substance use disorders	18 (36%)	0 (0%)	**0.002** ^b^
Mood disorders	38 (76%)	18 (90%)	0.186^b^
Anxiety disorders	31 (62%)	10 (50%)	0.357^b^
Physiological disturbances and physical factors	10 (20%)	2 (10%)	0.316^b^
Disorders of adult personality and behavior	21 (42%)	1 (5%)	**0.003** ^b^
Unspecified mental disorder	10 (20%)	2 (10%)	0.316^b^
Conduct disorder	6 (12%)	0 (0%)	0.105^b^
Borderline personality disorder	34 (68%)	0 (0%)	**< 0.001** ^b^
Number of BPD criteria	5.24 (1.91)	0.65 (0.81)	**< 0.001** ^a^
Depressive symptoms	*Not assessed*	23.60 (10.45)	-

Note. Significant results (*p* ≤ 0.05) in bold print

^a^ based on t-test

^b^ based on Chi^2^-test, variables are dummy-coded

**Fig. 1:**
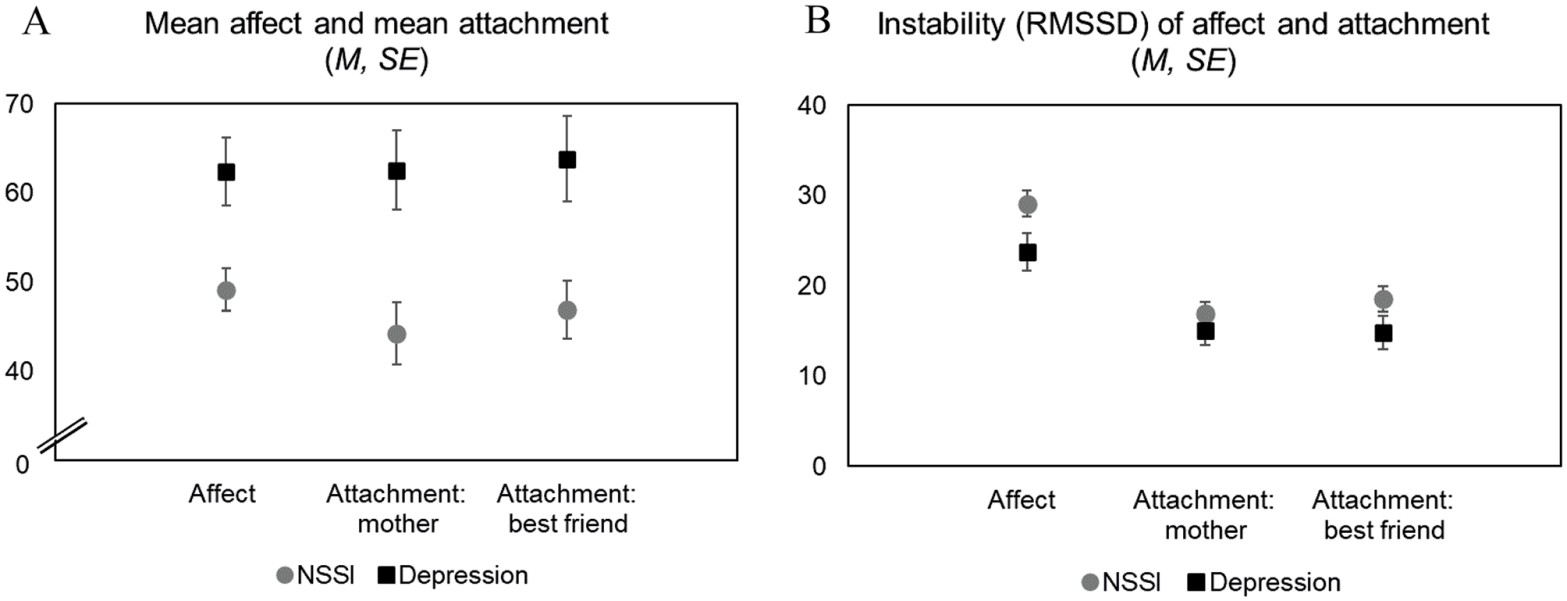
Group-averages and their standard errors (using analytic weights) of affect and attachment (**a**) and instability of affect and attachment (**b**) in adolescents with NSSI and the clinical comparison group. Panel A: higher values indicate more positive affect and more perceived closeness with the mother/best friend. Panel B: higher values indicate more instability

Figure 1, *panel A* and results from the regression models (Table 2) show that adolescents with NSSI reported less positive affect and poorer attachment to the mother and the best friend than depressed adolescents without NSSI. The group differences were significant (*p* ≤ 0.05) when adjusting for gender, which had no significant main effect on the outcomes. On average, positive affect was 13 points lower in the NSSI group (standardized regression coefficient from adjusted model: *β* = − 0.35), and ratings of one’s attachment to the mother and the best friend were 18 (*β* = − 0.38) and 17 points (*β* = − 0.35) lower, respectively.

**Table 2:** Results from regression models, outcomes: affect and attachment; models using analytic weights

Outcomes/predictors	Model 1			Model 2		
	b^a^	95% CI	*p*	b^a^	95% CI	*p*
**Affect**						
NSSI (ref.: depression)	-13.21	-21.83 to -4.59	**0.003**	-13.33	-22.19 to -4.46	**0.004**
Female (ref.: male)				0.64	-9.26 to 10.55	0.897
**Attachment: mother**						
NSSI (ref.: depression)	-18.34	-30.10 to -6.58	**0.003**	-19.90	-31.85 to -7.95	**0.001**
Female (ref.: male)				8.70	-4.65 to 22.05	0.198
**Attachment: best friend**						
NSSI (ref.: depression)	-16.96	-28.31 to -5.61	**0.004**	-17.50	-29.15 to -5.85	**0.004**
Female (ref.: male)				3.00	-10.01 to 16.03	0.646

Note. Significant results (*p* ≤ 0.05) in bold print

^a^ Unstandardized coefficient

Figure 1, *panel B* and results from the regression models (Table 3) show that adolescents with NSSI experienced more affective instability than those in the depression group. The group difference was significant (*p* ≤ 0.05) when adjusting for gender and the individual mean score of affect (*β* = 0.27). No group differences emerged regarding instability of attachment to the mother or best friend. Overall, the findings from the analyses reported here were replicated in supplementary analyses without analytic weights (see supplementary Tables S1 and S2). An exception was that the unadjusted association (i.e., in a model excluding gender and the individual mean level of affect) between group and affective instability was not significant (*p* = 0.057).

**Table 3:** Results from regression models, outcomes: instability of affect and attachment (RMSSD); models using analytic weights

Outcomes/predictors	Model 1			Model 2			Model 3		
	b^a^	95% CI	*p*	b^a^	95% CI	*p*	b^a^	95% CI	*p*
**RMSSD affect**									
NSSI (ref.: depression)	5.38	0.37 to 10.38	**0.036**	5.76	0.66 to 10.87	**0.027**	6.26	0.79 to 11.73	**0.025**
Female (ref.: male)				-2.40	-8.08 to 3.27	0.401	-2.40	-8.11 to 3.30	0.403
Mean affect							0.04	-0.10 to 0.18	0.600
**RMSSD attachment: mother**									
NSSI (ref.: depression)	1.93	-2.18 to 6.04	0.351	1.99	-2.22 to 6.20	0.349	0.98	-3.56 to 5.53	0.667
Female (ref.: male)				-0.35	-5.03 to 4.33	0.882	0.06	-4.67 to 4.78	0.981
Mean attachment: mother							-0.50	-0.14 to 0.04	0.250
**RMSSD attachment: best friend**									
NSSI (ref.: depression)	3.68	-1.07 to 8.43	0.126	4.08	-0.76 to 8.91	0.097	2.16	-2.84 to 7.16	0.392
Female (ref.: male)				-2.45	-7.82 to 2.92	0.366	-2.13	-7.34 to 3.10	0.419
Mean attachment: best friend							-0.11	-0.21 to -0.01	**0.029**

Note. Significant results (*p* ≤ 0.05) in bold print

^a^ Unstandardized coefficient

Our exploration of gender differences needs to be considered with caution, given that these models were likely statistically underpowered due to the small size of our sample. Effects of interaction terms between group and gender (added in version 2 of each regression model) were non-significant (mean outcomes: *p* = 0.663 for affect, *p* = 0.437 for attachment to mother, *p* = 0.309 for attachment to best friend; instability outcomes: *p* = 0.193 for affect, *p* = 0.940 for attachment to mother, *p* = 0.487 for attachment to best friend). However, separate models based on the male and female sub-samples indicated some gender-specific links (detailed results are provided in the supplementary material, Table S3). In brief, the NSSI groups consistently reported less positive affect than the depression groups, whereas a significant (*p* ≤ 0.05) group difference between the NSSI and depression groups regarding affective instability emerged in the male sub-sample only, despite the rather small sample size. An association between NSSI and poorer attachment was observed only in the female sub-sample, whereas for attachment instability, no group differences emerged in any of the sub-samples.

### Sensitivity analysis: symptom severity and instability in the depression group

**Fig. 2:**
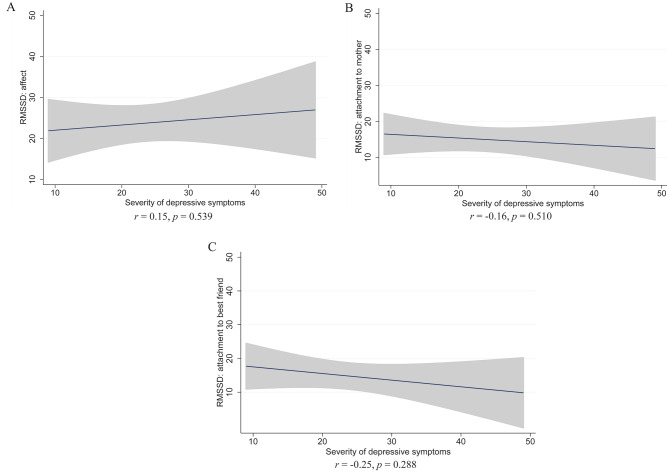
Regression lines and 95% confidence intervals for the associations between affective and attachment instability and severity of depressive symptoms in the depression group. Correlation coefficients and *p*-values are provided below the plots

Our general assumption was that differences between the NSSI and depression groups emerge due to differential underlying psychopathology. However, the observed group differences could also be due to lower levels of psychiatric impairments among participants in the depression group. To investigate this further, we carried out a sensitivity analysis of the associations between the severity of depressive symptoms and instability of (a) affect, (b) attachment to the mother, and (c) attachment to the best friend in the depression group. The results revealed that instability was generally independent from depressive symptoms severity (Fig. 2), which supports the notion that group differences were linked to differential types of psychopathologies.

## Discussion

Affective instability has been associated with adolescent NSSI [e.g., 17, 20], but also with a variety of psychiatric disorders [25], including depression [5]. Investigations of the specificity of affective instability to NSSI have been based on adult samples with BPD [23, 24], which often involves NSSI, whereas respective data from adolescents were missing. Our findings provide support for the hypothesis that adolescents with NSSI experience more affective instability than depressed adolescents not engaging in NSSI. Adolescents with NSSI also experienced less positive affect and lower attachment levels than depressed adolescents, although attachment instability was not specific to NSSI in our sample. Our preliminary explorations of gender differences suggest that the strength of the association between instability and NSSI may differ between male and female adolescents.

Our finding that adolescents with NSSI experience more negative and instable affect and poorer interpersonal attachment than those in the depression only group confirms that the psychopathology underlying NSSI is largely characterized by emotional and interpersonal dysfunction [4, 9], and that NSSI may serve as a marker of psychopathological burden in clinical samples. Moreover, our results strengthen conclusions from prior investigations comparing adolescents with NSSI to healthy control groups. Specifically, our findings support the notion that affective instability is a core feature of the psychopathology underlying NSSI [17], which implies that this psychopathology overlaps with what has been suggested as dimensional borderline personality pathology [12]. The results from our sensitivity analyses further support the assumption that the type of underlying psychopathology is more important than symptom severity in explaining the observed group differences in affective instability. Importantly, the group engaging in NSSI generally had higher rates of personality disorders and substance use, which might also be expressions of their high levels of emotional dysregulation. While such comorbidities are common among adolescents with NSSI, future investigations based on larger samples and more selective recruitment could disentangle their specific associations with affective instability.

Our study adds to previous research that has been based on adult samples and provided mixed evidence regarding the specificity of affective instability to BPD, without addressing the specific role of NSSI. Like Trull et al.’s study [10], which indicated that daily life affective instability is specific to BPD, our study was based on a gender-diverse sample, included a comparison group with depressive symptoms (of note, Trull et al. had explicitly recruited a group with depression not fulfilling the affective instability criterion of BPD), and the intervals between assessments used to compute instability scores were relatively long (here: up to 150 min). By contrast, Santagelo et al.’s [24] and Kockler et al.’s [23] studies were based on female-only samples, included comparison groups with other disorders (e.g., post-traumatic stress disorder) and used shorter intervals between adjacent assessments for computing instability scores (e.g., 15 min). These latter studies did not support specificity assumptions about affective instability. Together, the previous and our new evidence suggest that research is needed to systematically compare specific disorders, genders, and conceptualizations/measures of instability to better understand to what extent affective instability may be specific to borderline personality pathology or NSSI. Finally, age-graded associations between affective instability and psychopathology [44] need to be considered systematically in future research.

Contrary to our hypotheses, attachment instability was not uniquely associated with adolescent NSSI, indicating that moment-to-moment interpersonal dynamics were similar in the depression group. A possible explanation could be that the NSSI group primarily included adolescents who engaged in NSSI for emotion regulation and less so for interpersonal regulation, although the latter is also a possible function of NSSI among adolescents [4]. Furthermore, previous evidence has shown that emotion dysregulation can mediate links between interpersonal problems and NSSI [6], but our null finding could indicate that attachment and affective instability are often independent from each other and attachment instability may trigger NSSI only if/when it is also linked with affective instability. In other words, attachment instability alone may not be considered a ‘driving force’ behind NSSI. It is also possible that experiences of unstable attachment with the mother and best friend is similar in adolescent NSSI and depression (i.e., a transdiagnostic factor), and future research is needed to examine whether this holds for other attachment figures, such as intimate partners, other caregivers, peers, and role models.

Our explorations of gender-specific links between NSSI and affect and attachment need to be interpreted with caution due to the small group sizes and low statistical power in the moderation model. They could indicate that inter- and intra-personal dynamics play a differential role in male versus female NSSI. Although we did not find evidence of a moderating effect of gender in the reported associations, the link between affective instability and NSSI was mainly driven by the male subsample, whereas low levels of attachment were associated with NSSI especially in females. Other research has documented gender-specific pathways to psychopathology, including NSSI, and an elevated sensitivity to interpersonal stressors among females [45–47]. The preliminary results from our study may encourage more research on gender as a potential moderator of associations between daily intra- and interpersonal instabilities and adolescent NSSI, which have not been investigated previously.

Overall, our findings have implications for clinical practice. Although mitigating affective instability is likely important for many adolescent patients with disorders not involving NSSI, it should become a major treatment target as soon as comorbid NSSI is observed. Highly relevant tools come especially from the dialectical behavioral therapy framework [48, 49]. To be able to use NSSI as an identifier of a patient’s urgent need for affect stabilization, therapists should engage in psychoeducation, provide their patients with tools that help them observe and interpret their own emotions and behaviors, and create an atmosphere where patients feel safe disclosing NSSI if/when it has occurred.

In and outside of the clinical context, understanding adolescent NSSI as an expression of severe affective instability may help families, youth, and professionals put the behavior into a developmental perspective—especially because NSSI often peaks in adolescence and then declines [12, 29]. While NSSI may often be a temporary phenomenon related to emotional ups and down in adolescence, recent research has identified symptom shifts to other risky behaviors, such as substance use, as indication of emerging or persisting borderline symptomatology [50]. It may be advisable for clinicians and researchers to explore whether persistent affective instability could be a driving force behind such symptom shifts during adolescent development.

### Strengths, limitations, and directions for future research

A main strength of our study is the use of EMA to assess moment-to-moment affective and attachment dynamics in daily life, which facilitates an ecologically valid representation of individual experiences and is not prone to recall bias [51]. The frequency of prompts was relatively high compared to other studies, following recommendations for the assessment of affective instability in BPD [52]. However, data were collected only on weekends, given that smartphone use was restricted at schools. Consequentially, shifts of affect and attachment in response to, for example, social experiences at school and thereafter could not be considered. Other studies have demonstrated that adolescent behavior and emotional states can vary across weekdays and social environments, such as home versus school [18]. Whenever possible, future research should thus consider assessments of affect and attachment on schooldays, if not restricted by authorities.

It is also important to note that we focused on the adolescents’ relationships with their mothers and best friends only. Other relationships that gain importance during the adolescent period include those with intimate partners. Previous evidence suggests that NSSI may be more closely linked with romantic stress than stress experienced in other relationships when adolescents reach advanced pubertal stages [53]. Future studies are needed to examine the links between instability experienced in romantic relationships and NSSI and related borderline personality pathology versus other disorders. On a more general note, future research using EMA is needed to illuminate further the associations between instability of attachment and affect and their joint impact on NSSI.

An additional strength of our study is the comparison of two clinical groups that are well-defined and distinguished regarding psychiatric diagnoses and symptoms. This study design overcomes major weaknesses of prior research on the features of NSSI and associated BPD symptoms, where the overlap between NSSI and other disorders could often not be systematically considered [17]. However, the unbalanced group sizes in our study may limit the robustness of our results. Moreover, both groups were characterized by considerable comorbidity of psychiatric diagnoses that were not at the core of our investigation. To facilitate in-depth sub-group comparisons, including between groups affected by different forms and levels of (comorbid) psychopathology, more research with larger samples, balanced group sizes, and selective recruitment is needed. However, the sample studied here shows high representativeness of a common clinical sample in this age group. In the absence of strict inclusion criteria regarding clinical comorbidity, the generalizability of our findings represents a strength.

It is possible that some adolescents in the depression-without-NSSI-group falsely negated NSSI, for example due to social desirability [54]. However, the risk of inaccurate reporting of NSSI may be relatively low in a clinical setting with in-depth diagnostic assessments, which have been utilized in our study.

Finally, we were able to include male and female participants, which is rare in clinical research on NSSI [35]. Although the interaction effect between group and gender was non-significant in the present study, it is noteworthy that the association between NSSI and affective instability was mainly driven by the male sub-sample. A caveat of our study is that the analysis of interaction effects may have been statistically underpowered, given the small group size especially of the male sub-sample. Furthermore, although we were able to identify links between NSSI and affective instability, the size of our sample and the unbalanced gender ratio may have limited statistical power to detect potential other associations (e.g., potential links between NSSI and attachment instability). However, limitations of power and precision must be weighed against the value of providing first insights into largely understudied groups of adolescent patients [55]. Our study may encourage future research endeavors aiming to recruit larger and gender-diverse clinical comparison groups to increase the precision of estimates, evaluate the generalizability of our findings, and investigate potential gender-differences further.

## Conclusion

NSSI is a transdiagnostic phenomenon that co-occurs with a variety of psychiatric disorders [5, 15]. Comparisons between adolescents with NSSI and clinical control groups without NSSI regarding symptom types and severity are needed to sharpen diagnostic criteria and identify the individual relevance of specific treatment targets. However, such comparisons are rare. Using innovative research methodology to assess moment-to-moment changes of affect and attachment, we compared two well-distinguished clinical groups of adolescents with and without NSSI. The findings suggest that affective dynamics may play a unique role in adolescent NSSI. Thus, our findings highlight the importance of targeting affective instability in psychiatric treatments of adolescent patients who present with (comorbid) NSSI. Future research based on larger, gender-diverse samples is needed to replicate our findings and further investigate the mechanisms underlying the links between affective and attachment instability and NSSI.

## Supplementary Information

Below is the link to the electronic supplementary material.

Supplementary Material 1

## Data Availability

The datasets used and/or analysed during the current study are available from the corresponding author on reasonable request.
