# Supplementary material for: How specific are affective and attachment instability to adolescents with non-suicidal self-injury? A comparison with adolescents with major depression
Source: Borderline Personal Disord Emot Dysregul. 2026 Feb 12;13:6. doi: 10.1186/s40479-026-00336-4 (PMC12997848; doi:10.1186/s40479-026-00336-4)
Supplement: Supplementary file 1 — Supplementary Material 1 [file 40479_2026_336_MOESM1_ESM.docx]

**Online Supplement**

How specific are affective and attachment instability to adolescents with non-suicidal self-injury?

A comparison with adolescents with major depression

Annekatrin Steinhoff, Julian Koenig, Julia Blanke, Philip Santangelo, Ulrich Ebner-Priemer, & Michael Kaess

***Table S1.*** Results from regression models, outcomes: affect and attachment; unweighted.

| **Outcomes/predictors** | **Model 1** |  |  | **Model 2** |  |  |
| --- | --- | --- | --- | --- | --- | --- |
|  | ***b***^a^ | **95% CI** | ***p*** | ***b***^a^ | **95% CI** | ***p*** |
| **Affect** |  |  |  |  |  |  |
| NSSI (ref.: depression) | -13.51 | -22.25 to -4.77 | **0.003** | -13.75 | -22.81 to -4.68 | **0.003** |
| Female (ref.: male) |  |  |  | 1.14 | -9.10 to 11.37 | 0.825 |
| **Attachment: mother** |  |  |  |  |  |  |
| NSSI (ref.: depression) | -17.99 | -30.30 to -5.67 | **0.005** | -20.01 | -32.62 to -7.41 | **0.002** |
| Female (ref.: male) |  |  |  | 9.65 | -4.58 to 23.88 | 0.181 |
| **Attachment: best friend** |  |  |  |  |  |  |
| NSSI (ref.: depression) | -16.05 | -27.87 to -4.23 | **0.009** | -16.60 | -28.84 to -435 | **0.009** |
| Female (ref.: male) |  |  |  | 2.62 | -11.21 to 16.44 | 0.707 |

*Note.* Significant results (p ≤ 0.05) in bold print.

^a^ Unstandardized coefficient

***Table S2.*** Results from regression models, outcomes: instability of affect and attachment (RMSSD); unweighted.

| **Outcomes/predictors** | **Model 1** |  |  | **Model 2** |  |  | **Model 3** |  |  |
| --- | --- | --- | --- | --- | --- | --- | --- | --- | --- |
|  | ***b***^a^ | **95% CI** | ***p*** | ***b***^a^ | **95% CI** | ***p*** | ***b***^a^ | **95% CI** | ***p*** |
| **RMSSD affect** |  |  |  |  |  |  |  |  |  |
| NSSI (ref.: depression) | 5.43 | -0.18 to 11.03 | 0.057 | 5.87 | 0.75 to 11.67 | **0.047** | 7.32 | 1.18 to 13.47 | **0.020** |
| Female (ref.: male) |  |  |  | -2.11 | -8.65 to 4.44 | 0.522 | -2.23 | -8.74 to 4.28 | 0.497 |
| Mean affect |  |  |  |  |  |  | 0.11 | -0.05 to 0.26 | 0.178 |
| **RMSSD attachment: mother** |  |  |  |  |  |  |  |  |  |
| NSSI (ref.: depression) | 2.03 | -2.74 to 6.80 | 0.398 | 2.03 | -2.92 to 6.97 | 0.417 | 1.94 | -3.41 to 7.29 | 0.472 |
| Female (ref.: male) |  |  |  | 0.03 | -5.56 to 5.62 | 0.992 | 0.07 | -5.64 to 5.78 | 0.980 |
| Mean attachment: mother |  |  |  |  |  |  | -0.00 | -0.10 to 0.09 | 0.928 |
| **RMSSD attachment: best friend** |  |  |  |  |  |  |  |  |  |
| NSSI (ref.: depression) | 3.73 | -1.65 to 9.11 | 0.171 | 4.07 | -1.50 to 9.64 | 0.149 | 2.20 | -3.53 to 7.93 | 0.446 |
| Female (ref.: male) |  |  |  | -1.63 | -7.92 to 4.66 | 0.607 | -1.33 | -7.48 to 4.82 | 0.667 |
| Mean attachment: best friend |  |  |  |  |  |  | -0.11 | -0.22 to -0.00 | **0.041** |

*Note.* Significant results (p ≤ 0.05) in bold print.

^a^ Unstandardized coefficient

***Table S3.*** Results from separate regression models for males and females (weighted).

| **Outcomes/predictors** | **Male** |  |  | **Female** |  |  |
| --- | --- | --- | --- | --- | --- | --- |
|  | ***b***^a^ | **95% CI** | ***p*** | ***b***^a^ | **95% CI** | ***p*** |
| **Affect** |  |  |  |  |  |  |
| NSSI (ref.: depression) | -16.54 | -28.67 to -4.41 | **0.012** | -12.14 | -23.28 to -0.99 | **0.033** |
| **Attachment: mother** |  |  |  |  |  |  |
| NSSI (ref.: depression) | -12.18 | -40.15 to 15.79 | 0.361 | -22.76 | -36.48 to -9.04 | **0.002** |
| **Attachment: best friend** |  |  |  |  |  |  |
| NSSI (ref.: depression) | -7.68 | -28.58 to 13.23 | 0.439 | -21.14 | -35.24 to -7.04 | **0.004** |
| **RMSSD: affect** |  |  |  |  |  |  |
| NSSI (ref.: depression) | 11.24 | 4.93 to 17.56 | **0.002** | 3.72 | -2.67 to 10.11 | 0.248 |
| **RMSSD: Attachment to mother** |  |  |  |  |  |  |
| NSSI (ref.: depression) | 1.73 | -4.31 to 7.76 | 0.545 | 2.09 | -3.19 to 7.37 | 0.431 |
| **RMSSD: Attachment to best friend** |  |  |  |  |  |  |
| NSSI (ref.: depression) | 6.86 | -1.99 to 15.70 | 0.117 | 3.04 | -2.82 to 8.90 | 0.303 |

*Note.* Significant results (p ≤ 0.05) in bold print.

^a^ Unstandardized coefficient
